# Supplementary material for: Pathophysiological Significance of Neutrophilic Transfer RNA-Derived Small RNAs in Asymptomatic Moyamoya Disease
Source: Cells. 2021 May 1;10(5):1086. doi: 10.3390/cells10051086 (PMC8147334; doi:10.3390/cells10051086)

2D Structure | **Local AU** | **Position**

```
484                        512
                  8mer-1a
5'-gatacaGA-AACTACAACCCCGACGGAAAa-3' utr
        ||  |||:||  |  |  | ||||||
3'-uuugcaCUAUUGGUGAU-GUGAUGCCUUUg-5' tRF
               16151413     7 6 5 4 3 2
             3' Pairing       Seed
```

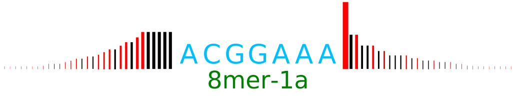

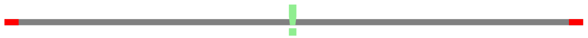

Supplement: Supplementary file 1 [file cells-10-01086-s001.zip › cells-1149161 3rd supplementary materials/Data Supplements/Data Supplement V.zip!/Data supplement 6-mRNAs targets of tsRNAs/tsRNA-Val-AAC-017/Diagrams/tRF-Val-AAC-017_vs_HBMT00000920938(CATG00000056069.1).pdf]
